# Attenuation of Diabetic Nephropathy in Otsuka Long-Evans Tokushima Fatty (OLETF) Rats with a Combination of Chinese Herbs (Tangshen Formula)

**DOI:** 10.1155/2011/613737

**Published:** 2011-01-17

**Authors:** Haojun Zhang, Ping Li, Frank J. Burczynski, Yuewen Gong, Patrick Choy, Hong Sha, Jing Li

**Affiliations:** ^1^Department of Pharmacology, Institute of Clinical Medical Sciences, China-Japan Friendship Hospital, No. 2 Yinghua Dongjie, Hepingli, Beijing 100029, China; ^2^Faculty of Pharmacy, University of Manitoba, 750 McDermot Avenue, Winnipeg, MB, Canada R3E 0T5; ^3^Faculty of Medicine, University of Manitoba, 750 McDermot Avenue, Winnipeg, MB, Canada R3E 0T5; ^4^Dongzhimen Hospital, Beijing University of Chinese Medicine, No. 5 Hai Yun Cang, Dongcheng District, Beijing 100700, China

## Abstract

Diabetic nephropathy is one of the most significant microvascular complications in patients with type 2 diabetics. The concise mechanism of diabetic nephropathy is unknown and there is no successful treatment. The objective of study was to investigate effects of Chinese herbs (Tangshen Formula) on diabetic nephropathy in Otsuka Long-Evans Tokushima Fatty (OLETF) rats. OLETF rats and LETO rats were divided into four groups: LETO control, OLETF diabetics, OLETF diabetics treated with Tangshen Formula, and OLETF diabetics treated with Monopril. Body weight, blood glucose, and 24 h urinary proteins were measured once every four weeks. Blood samples and kidney tissues were obtained for analyses of total cholesterol, triglyceride, whole blood viscosity, plasma viscosity, and pathohistological examination at 36 and 56 weeksrespectively. Untreated OLETF rats displayed diabetic nephropathy over the study period. Treatment of OLETF rats with Tangshen Formula attenuated the increases in blood glucose, body weight, 24 h urinary protein content, serum total cholesterol, whole blood viscosity and plasma viscosity at certain time. Treatment with Tangshen Formula also reduced glomerulosclerotic index and interstitial fibrotic index seen in OLETF rats. In conclusion, Tangshen Formula could attenuate the development of diabetic nephropathy in OLETF rat diabetic model.

## 1. Introduction

Diabetic nephropathy (DN) is one of the most important microvascular complications associated with type 2 diabetic patients and has emerged as a leading cause of the end-stage renal disease in developed countries [1, 2]. DN is characterized by structural abnormalities of kidney including hypertrophy of both glomerular and tubular elements, increase in the thickness of glomerular basement membranes, and progressive accumulation of extracellular matrix components, eventually leading to proteinuria and renal failure [3]. Despite implementation of intensive glycemic and antihypertensive control, DN remains an important clinical problem [2] and new therapeutic agents are needed for the treatment of this condition.

A useful model of the human type II diabetes mellitus is the Otsuka Long-Evans Tokushima Fatty (OLETF) rat. This model is characterized by the development of late-onset hyperglycemia, mild obesity, and the associated diabetic complications. At 22 weeks of age the OLETF rats develop overt albuminuria and at 30 weeks, OLETF showed significant proteinuria and morphological changes in the kidney such as glomerular hypertrophy and extracellular matrix expansion. At 54 weeks, OLETF rats developed advanced kidney injuries such as heavy proteinuria, diffuse glomerulosclerosis, nodular lesions and severe tubulointerstitial fibrosis, which are resemble to late stage of human DN. Thus, the OLETF rat is considered to be a useful animal model to study type 2 diabetic nephropathy [4–6].

In China, traditional herbal medicines have been widely used for the treatment of diabetes and its complications for thousands of years. Recently they have received worldwide attention and are becoming promising sources of new therapeutic agents for diabetic nephropathy [7]. One such medicine is the Tangshen Formula. It is composed of several herbs and formulated according to Traditional Chinese Medicine teachings. In our clinical study, we found that Tangshen Formula had improvement effects on proteinuria and hyperlipidemia in DN patients [8]. However, to date, there is little experimental evidence to explain these effects. To this end, we investigated the effect of the Tangshen Formula treatment on renal function, morphological changes, serum lipid, and hemorheology in the OLETF rat.

## 2. Materials and Methods

### 2.1. Animals and Experimental Design

Four-week-old male OLETF rats and age-matched Long-Evans Tokushima Otsuka (LETO) rats were kindly provided by Tokushima Research Institute (Otsuka Pharmaceutical, Tokushima, Japan). All rats were housed at 22 ± 3°C and 50 ± 10% humidity using a 12 h light/dark cycle. All animals were given free access to standard rat chow and water. Four groups of rats were prepared at 12 weeks of age:

1. non-diabetic LETO rats given distilled water (no treatment, *n* = 20, LETO control),
2. diabetic OLETF rats given water (no treatment, *n* = 15, OLETF control),
3. diabetic OLETF rats administered Tangshen Formula (1.60 g/kg body weight/day, *n* = 15, OLETF + TSF),
4. OLETF rats administered Monopril (Bristol-Myers Squibb, USA, 0.833 mg/kg body weight/day, *n* = 15, OLETF + monopril).

All drugs were dissolved in distilled water and administered once daily by gastric gavage. The powdered formulation of Tangshen Formula was composed of radix astragali (24 g), radix rehmanniae (15 g), radix notoginseng (2 g), prepared radix et rhizome rhei with wine (8 g), fructus aurantii (6 g), fructus corni (11 g) and ramulus euonymi (12 g) (Tianjiang Pharmacology Co. Ltd, China). The experimental extraction procedure was identical to that used for the clinical preparation of the formula. Briefly, herbs were well mixed and soaked in distilled water for 30 min, boiled in 10 volumes of water (v/w) for 1 h, and extracted twice. The extract was filtrated and condensed to the concentration of 1 g/ml and processed to powder by spray drying. The volume administered to the animals was calculated to be 1 ml/100 g body weight [9]. 

Ten rats from the LETO control group and seven rats from the other three groups were sacrificed at 36 weeks of age. The remaining rats in each group were sacrificed at 56 weeks of age. The endpoints of experiment were determined according to pathological changes during life span of OLETF rats. Rats were sacrificed by intraperitoneal injection of chloral hydrate. Blood samples were then collected from the abdominal aorta, and kidneys were removed and separated into two pieces for histopathological examination. This study was approved by the Ethics Committee of China-Japan Friendship Institute of Clinical Medicine and performed in accordance with Guiding Principles for the Care and Use of Laboratory Animals.

### 2.2. Determination of Body Weight, Blood Sample and Urinary Protein

Body weight of rats was measured at 4-week intervals. Blood was sampled from the tail vein at 4-week intervals, and blood glucose levels measured by One Touch Ultrablood glucose monitoring system (LifeScan, USA). Rats were housed individually in metabolic cages (Fengshi, China) for 24-h urinary collection at 4-week intervals. 

Rats were sacrificed and aortic blood was collected and divided into 2 parts. One part of blood samples was collected into tubes without anticoagulant and centrifuged at 3000 g/min and 4°C for 15 min. Serum was separated and total cholesterol, triglycerides, urea nitrogen, creatinine, total protein, and albumin were measured using a CD-1600CS hematology analyzer (Abbott Labs, USA).

### 2.3. Measurement of Blood Viscosity

The other part of blood was collected into tubes containing Na_2_EDTA. Blood viscosity was measured at 37°C with a coaxial cylinder microviscometer (BV-100, China) at a shear rate of 0.945 s and 94.5 s followed by plasma viscosity determination after centrifugation.

### 2.4. Histological Examination of the Kidney

Sections of kidney tissue were removed and immediately fixed in 10% phosphate buffered formalin solution and embedded in paraffin. Sections (3-*μ*m) from each sample were cut and stained with periodic acid Schiff's stain for determination of glomerulosclerosis. The degree of glomerulosclerosis, defined as a thickening of the basement membrane and mesangial expansion, was evaluated as described previously [10]. In brief, the prepared kidney sections were observed under an Olympus BX51 light microscope (Olympus, Japan) at a magnification of ×400 with an Olympus DP70 digital imaging system (Olympus, Japan). Forty glomeruli in each kidney were graded in accordance with their severity of glomerular damage (0, normal; 1, slight glomerular damage, the mesangial matrix and/or hyalinosis with focal adhesion involving <25% of the glomerulus; 2, sclerosis of 26–50%; 3, sclerosis of 51–75%; and 4, sclerosis of >75% of the glomerulus). The glomerulosclerotic indices were calculated using the formula: glomerulosclerotic index = (1 × *n*1) + (2 × *n*2) + (3 × *n*3) + (4 × *n*4)/*n*0 + *n*1 + *n*2 + *n*3 + *n*4, where *nx* is number of glomeruli in each grade of glomerulosclerosis. The observer was blinded to all tissue samples.

Tubulointerstitial and vascular damage was assessed on periodic acid Schiff's-stained paraffin sections at a magnification of ×100 using a similar scoring system (0–4) as described previously in detail [11].

### 2.5. Statistics

Data are presented as mean ± SEM unless stated otherwise. Significant differences between groups were compared using ANOVA with *P* < .05 being considered statistically significant. 

## 3. Results

**Figure 1:**
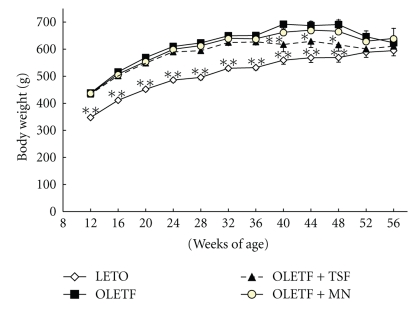
Effects of Tangshen Formula and Monopril on body weight in the rats during the 56 week treatment period. Data represent mean ± SEM; *P < .05 and **P < .01 compared to control OLETF rats.

Body weight of OLETF rats was heavier than that of LETO rats from 12 to 36 weeks (Figure 1). However from weeks 40 to 48, body weight of OLETF rats treated with Tangshen Formula was not significantly different as compared with that of LETO rats, but it was significantly different than that of OLETF rats treated with water and Monopril. Moreover, at weeks 52 and 56, no significant difference of body weight wad observed among all these groups. 

**Figure 2:**
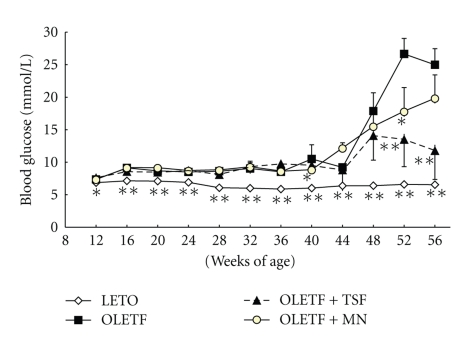
Effect of Tangshen Formula and Monopril on blood glucose in the rats during the 56 week treatment period. Data represent mean ± SEM; *P < .05 and **P < .01 compared to control OLETF rats.

Blood glucose levels for the LETO control rats did not change throughout the study period (Figure 2). Blood glucose of the age-matched OLETF rats was statistically higher than those of LETO rats throughout the study period (Figure 2). Compared to control OLETF rats, blood glucose of OLETF rats treated with Tangshen Formula exhibited statistically lower blood glucose at 52 and 56 weeks of age. Blood glucose of OLETF rats treated with Monopril also became elevated during the study period. Except at weeks 40 and 52, blood glucose in this group was not significantly different from the OLETF control group (Figure 2).

**Figure 3:**
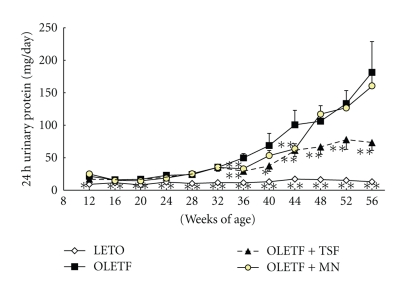
Effects of Tangshen Formula and Monopril on 24-h urinary protein in the rats during the 56 week treatment period. Data represent mean ± SEM; *P < .05 and **P < .01 compared to control OLETF rats.

Urinary protein in all OLETF rats gradually and continually increased during the entire study period compared with levels in the LETO rats that remained unchanged (Figure 3). Rats treated with Tangshen Formula had significantly lower levels of 24 h urinary protein compared to the control OLETF rats starting at 36 weeks of age. Urinary protein in rats treated with Monopril was only significantly lower than that the OLETF control group at weeks 24, 36, and 44 (Figure 3).

 There was no statistical difference in whole blood viscosity at 0.945 s and 94.5 s among the four groups at 36 weeks of age. However, whole blood viscosity for control OLETF rats was markedly higher than those of LETO rats at 56 weeks of age (Table 1). These two indices were significantly lower in OLETF rats treated with Tangshen Formula compared with those of the control (untreated) OLETF rats. Monopril had no effect on whole blood viscosity (Table 1).

Plasma viscosity of control OLETF rats was markedly increased compared with the LETO rats at 36 and 56 weeks of age. Plasma viscosity of the Tangshen Formula treated OLETF rats were significantly lower than that of the untreated rats at weeks 36 and 56 (Table 1). 

There was no statistical difference in total cholesterol between control OLETF rats and LETO rats at 36 weeks of age (Table 2). Compared with the LETO rats, however, total cholesterol was significantly increased (*P* < .01) in untreated OLETF rats at 56 weeks of age. Triglyceride levels in untreated OLETF rats were markedly increased compared to the LETO rats at 36 and 56 weeks of age (*P* < .01). Serum total protein and albumin of control OLETF rats were similar to LETO rats at 36 weeks of age; however, they were statistically lower at 56 weeks of age. There was no difference in urea nitrogen levels between LETO and control OLETF rats at 36 weeks of age but urea nitrogen levels of control OLETF rats were higher than those of LETO rats at 56 weeks of age. Serum creatinine levels in untreated OLETF rats were significantly lower than in LETO rats at both 36 and 56 weeks of age (Table 2). This finding suggests that glomerular perfiltration in diabetic kidneys may be taking place. Tangshen Formula improved cholesterol, total protein and albumin of OLETF rats at 56 weeks of age (*P* < .01) while triglyceride levels of OLETF rats were significantly decreased by Monopril at 36 weeks of age (*P* < .05).

**Figure 4:**
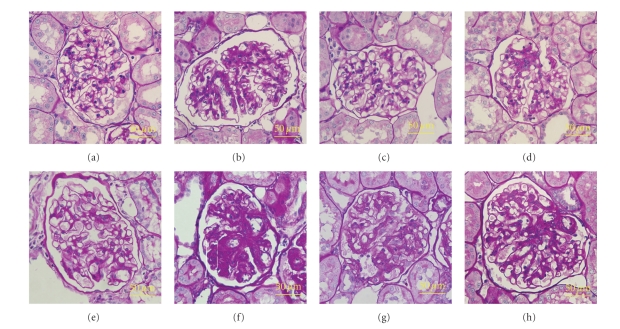
Periodic acid-Schiff staining of glomeruli of the rats at 36 weeks of age: (a) control LETO group; (b) control OLETF group, (c) OLETF + TSF group, (d) OLETF+Monopril group; and at 56 weeks of age: (e) control LETO group; (f) control OLETF group, (g) OLETF + TSF group, (h) OLETF + Monopril group, in diabetic OLETF rats, mesangial expansion accompanied by an accumulation of extracellular matrix and glomerular capillary wall thickening occurs at 36 weeks of age. Diffuse glomerularsclerosis and mesangial matrix expansion happened at 56 weeks of age. These glomerular changes are ameliorated by TSF and monopril treatment. Pictures is at the magnification of ×400.

**Figure 5:**
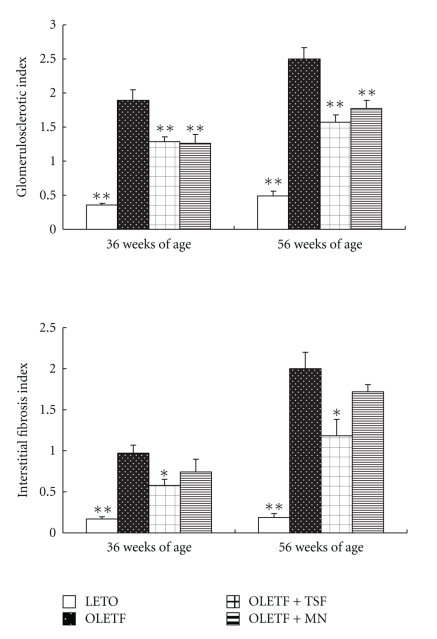
Effect of Tangshen Formula and Monopril on glomerulosclerotic index and interstitial fibrotic index in the rats at 36 and 56 weeks of age. Data represent as mean ± SEM; *P < .05, *P < .01 versus control OLETF rats.

Glomerular lesions in OLETF rats were characterized by hyalinosis, thickening of the basement membrane, mesangial expansion and sclerotic lesions. These pathological changes at 56 weeks of age were more severe than those at 36 weeks of age. Interstitial fibrosis in OLETF rats was focal and mild at 36 weeks of age while at 56 weeks of age severe changes occurred that included protein cast, tubular dilation or atrophy, and infiltration of inflammatory cells. Treatment with Tangshen Formula and Monopril significantly suppressed glomerulosclerosis in OLETF rats. Interstitial fibrotic lesions in OLETF rats were significantly improved by treatment with Tangshen Formula at both 36 and 56 weeks of age (Figure 4). Glomerulosclerosis index and interstitial fibrosis index in untreated OLETF rats increased significantly compared with those in LETO rats at 36 and 56 weeks of age.  Figure 5 shows that the Tangshen Formula and Monopril treatment both attenuated the increase in glomerulosclerosis index at 36 and 56 weeks while only Tangshen Formula was able to attenuate the increase in the interstitial fibrosis.

## 4. Discussion

In recent years the number of people suffering from the end-stage renal disease has increased worldwide. Approximately one third of ESRD results from diabetic nephropathy (DN). Effective treatment of DN is an important challenge to nephrologists today [12]. Animal studies show that the renin-angiotensin system (RAS) plays an important role in the pathogenesis of DN. Clinically, inhibition of the RAS by angiotensin converting enzyme inhibition and angiotensin receptor blockade (ARB) provide renoprotective benefits. Use of these drugs has been substantiated by both clinical trials and animal studies [13]. As a consequence, use of RAS antagonists has become the standard of care in diabetic patients with kidney disease and the utility of these agents continues to increase [14]. However, although it appears that such treatment slows the progression of DN development to ESRD, it does not stop or reverse the pathology. 

Traditional Chinese Medicine also exhibits beneficial effects on DN and is becoming a promising therapy [15, 16]. However, at this time, little experimental evidence is available to explain these effects. It is important, therefore, to further investigate the effects of Traditional Chinese Medicine and angiotensin converting enzyme inhibition on DN in experimental models of diabetes.

OLETF rats are widely used in many laboratories as a model of type 2 diabetes in humans. OLETF rats are characterized by late-onset hyperglycemia, a mild and chronic course of diabetes mellitus, and the associated complications of diabetes. Diabetes develops in almost all male OLETF rats by 25 weeks of age. As the disease progresses, renal complication becomes inevitable in this animal [4, 17]. 

Hyperglycemia is a critical factor in the development of diabetic nephropathy. It is associated with an increase in mesangial cell proliferation and hypertrophy, as well as increased matrix production and basement membrane thickening [18, 19]. The associated hyperglycemia might also upregulate VEGF expression in podocytes [20] which could markedly increase vascular permeability [21, 22]. Intensive glycemic control could, therefore, slow the development of diabetic nephropathy [23, 24]. In this investigation, Tangshen Formula had a significant effect on limiting the increase in blood glucose levels in OLETF rats at 52 weeks of age. In addition to its effects on blood glucose, Tangshen Formula also had a positive renoprotective effect. Improvement of the nephropathy observed in the Tangshen Formula treated group may be partially dependent on the effects Tangshen Formula has on glucose metabolism.

We observed an increase in total serum cholesterol in OLETF rats over the study period (approximately 70%, *P* < .01), while triglyceride levels increased by 13%. The increase in cholesterol is believed to be a compensatory production of lipids in the liver stimulated by hypoalbuminemia [25]. Circulating lipids are known to bind to extracellular matrix molecules where they undergo oxidation and increase the formation of reactive oxygen species (ROS) [26]. ROS reduce endothelium-derived vasodilators/growth inhibitors (such as prostacyclin and nitric oxide) and increase the formation of endothelium-derived vasoconstrictors/growth promoters (such as angiotensin II and endothelin-1) which are known to have significant renal pathophysiological effects [27, 28]. It is possible, therefore, that the associated hyperlipidemia could cause or contribute to renal injury [26, 29, 30]. 

We showed that Tangshen Formula and Monopril had beneficial effects on serum lipids. Total cholesterol was statistically lower in OLETF rats treated with Tangshen Formula at 56 weeks of age than control OLETF rats, while Monopril lowered triglyceride levels of OLETF rats at 36 weeks of age. It is not surprising that Monopril had a lipid-lowering effect since angiotensin converting enzyme inhibitors are known to lower serum total cholesterol and triglycerides in DN patients [31]. This reduction in triglycerides, however, was not sustained over the treatment period. The Chinese herbs, radix notoginseng, radix astragali and radix et rhizome rhei, all of which are present in the Tangshen Formula, have been reported to lower total serum cholesterol and triglyceride levels [32–34]. The present study showed that Tangshen Formula reduced only total cholesterol in OLETF rats. These differences may be due to dosage or drug administration since only one dose of the Tangshen Formula was used in this study. It is possible that the lipid-lowering effect of the Tangshen Formula may be associated with its renoprotective action.

Changes in blood rheology are an important factor that can lead to microvascular complications in diabetes. Hyperviscosity-related resistance to glomerular blood flow causes a compensatory elevation in glomerular hydrostatic pressure, which leads to an increase in protein permeability across the glomerular basement membrane. The increased viscosity may also lead to a cessation of glomerular blood flow and subsequent capillary thrombosis and glomerulosclerosis [35–38]. Our study showed that Tangshen Formula can reduce whole blood viscosity and plasma viscosity in OLETF rats, thereby potentially minimizing or preventing the pathological changes in the kidneys of OLETF rats.

**Figure 6:**
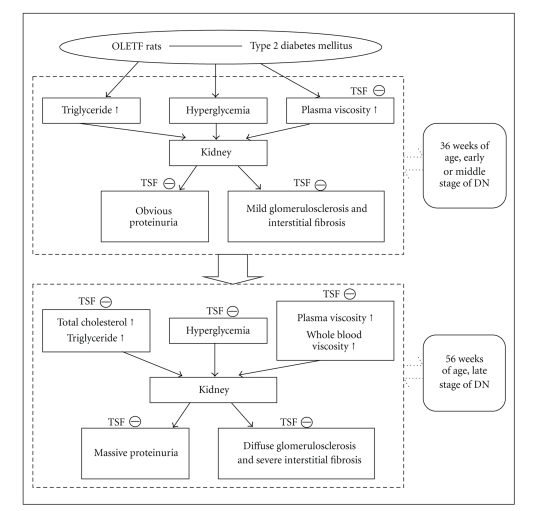
A diagram demonstrates the possible effect of Tangshen Formula in ameliorating diabetic nephropathy. At the early or middle state of DN, TSF improve proteinuria and mild renal pathological changes by decreasing plasma viscosity and at the late stage of DN, TSF alleviated massive proteinuria and severe glomerulosclerosis by regulating the levels of cholesterol, glucose and rheological changes. The definitions of abbreviations and symbols in the diagram are DN: diabetic nephropathy; TSF: Tangshen Formula.

The results from this study suggest that the beneficial effect of Tangshen Formula may be due to its ability to limit the development of hyperlipidemia as well as its beneficial effects on renal hemorheology in the OLETF rats. Tangshen Formula could reduce plasma viscosity at 36 weeks age, and at 56 weeks of age, Tangshen formula exhibits renoprotective effects by reduce the protein excretion and alleviated histological changes, the effects may be related to rectify the abnormality lipid metabolism, hyperglycemia and hemorheology disorder. This theory is illustrated in Figure 6. 

In conclusion, we found that Tangshen Formula attenuates the development of diabetic nephropathy in type 2 diabetic OLETF rats. Tangshen Formula may, therefore, represent a potentially new therapeutic strategy in the treatment of diabetic nephropathy in type 2 diabetes.

## Figures and Tables

**Table 1:** Rheological parameters in control and Tangshen Formula treated rats.

	36 weeks of age			56 weeks of age		

Groups	Whole blood	Whole blood	Plasma	Whole blood	Whole blood	Plasma
	viscosity	viscosity	viscosity	viscosity	viscosity	viscosity
	(0.945 s) (mPa·S)	(94.5 s) (mPa·S)	(mPa· S)	(0.945 s) (mPa·S)	(94.5 s) (mPa·S)	(mPa·S)
LETO	4.45 ± 0.14	17.90 ± 1.18	1.29 ± 0.03**	4.37 ± 0.14**	18.42 ± 1.04**	1.31 ± 0.03**
OLETF	4.67 ± 0.12	16.78 ± 0.87	1.42 ± 0.03	5.56 ± 0.15^##^	26.97 ± 1.38^##^	1.51 ± 0.06
OLETF + TSF	4.74 ± 0.28	14.05 ± 0.74	1.33 ± 0.02**	4.85 ± 0.06*	20.96 ± 2.22*^##^	1.35 ± 0.05*
OLETF + Monopril	4.67 ± 0.14	14.58 ± 1.00	1.37 ± 0.02	5.64 ± 0.38^#^	26.61 ± 1.10^##^	1.40 ± 0.05

Data represent mean ± SEM; *P < .05, **P < .01 versus control OLETF rats; ^#^ *P* < .05, ^##^ *P* < .01 compares the 36 and 56 weeks of age data groups.

**Table 2:** Biochemical indices of control and Tangshen Formula treated rats at 36 and 56 weeks of age.

	weeks	LETO	OLETF	OLETF + TSF	OLETF + Monopril
Total cholesterol (mmol/L)	36	2.71 ± 0.07	2.51 ± 0.15	2.59 ± 0.08	2.77 ± 0.30
	56	2.00 ± 0.06**^##^	4.27 ± 0.36^##^	2.21 ± 0.21**	3.94 ± 0.47
Triglyceride (mmol/L)	36	0.26 ± 0.04**	1.15 ± 0.12	1.01 ± 0.13	0.71 ± 0.05*
	56	0.21 ± 0.01**	1.30 ± 0.26	0.96 ± 0.07	1.07 ± 0.17^#^
Total protein (mmol/L)	36	60.30 ± 0.76	58.86 ± 1.08	59.29 ± 1.02	58.14 ± 1.06
	56	70.25 ± 0.99**^##^	67.17 ± 1.39^##^	73.17 ± 2.04**^##^	69.20 ± 1.39^##^
Albumin (mmol/L)	36	32.00 ± 0.42	30.86 ± 0.34	30.71 ± 0.52	30.57 ± 0.90
	56	32.06 ± 0.39*	28.62 ± 0.78^##^	31.83 ± 1.25**	29.26 ± 0.67
Urea nitrogen (mmol/L)	36	8.22 ± 0.26	7.53 ± 0.87	6.97 ± 0.50	8.52 ± 0.50
	56	5.28 ± 0.17**^##^	7.86 ± 1.02	6.60 ± 0.58	8.07 ± 1.12
Creatinine (*μ*mol/L)	36	89.22 ± 3.29**	68.00 ± 1.89	68.57 ± 1.19	70.86 ± 1.47
	56	69.75 ± 0.44**^##^	58.73 ± 3.01^##^	53.18 ± 1.69^##^	55.04 ± 2.60^##^

Data represent mean ± SEM, *P < .05, **P < .01 versus control OLETF rats; ^#^ *P* < .05, ^#^ *P* < .01 compares the 36 and 56 weeks of age data groups.
